# Predicting liver metastasis in pancreatic neuroendocrine tumors with an interpretable machine learning algorithm: a SEER-based study

**DOI:** 10.3389/fmed.2025.1533132

**Published:** 2025-05-01

**Authors:** Jinzhe Bi, Yaqun Yu

**Affiliations:** Department of Hepatobiliary and Pancreatic Surgery, Affiliated Hospital of Guilin Medical University, Guilin, China

**Keywords:** pancreatic neuroendocrine tumors, liver metastasis, machine learning, prediction, surveillance epidemiology and end results (SEER) database

## Abstract

**Background:**

Liver metastasis is the most common site of metastasis in pancreatic neuroendocrine tumors (PaNETs), significantly affecting patient prognosis. This study aims to develop machine learning algorithms to predict liver metastasis in PaNETs patients, assisting clinicians in the personalized clinical decision-making for treatment.

**Methods:**

We collected data on eligible PaNETs patients from the Surveillance, Epidemiology, and End Results (SEER) database for the period from 2010 to 2021. The Boruta algorithm and the Least Absolute Shrinkage and Selection Operator (LASSO) were used for feature selection. We applied 10 different machine learning algorithms to develop models for predicting the risk of liver metastasis in PaNETs patients. The model’s performance was assessed using a variety of metrics, including the area under the receiver operating characteristic curve (AUC), the area under the precision-recall curve (AUPRC), decision curve analysis (DCA), calibration curves, accuracy, sensitivity, specificity, F1 score, and Kappa score. The SHapley Additive exPlanations (SHAP) were employed to interpret models, and the best-performing model was used to develop a web-based calculator.

**Results:**

The study included a cohort of 7,463 PaNETs patients, of whom 1,356 (18.2%) were diagnosed with liver metastasis at the time of initial diagnosis. Through the combined use of the Boruta and LASSO methods, T-stage, N-stage, tumor size, grade, surgery, lymphadenectomy, chemotherapy, and bone metastasis were identified as independent risk factors for liver metastasis in PaNETs. Compared to other machine learning algorithms, the gradient boosting machine (GBM) model exhibited superior performance, achieving an AUC of 0.937 (95% CI: 0.931–0.943), an AUPRC of 0.94, and an accuracy of 0.87. DCA and calibration curve analyses demonstrate that the GBM model provides better clinical decision-making capabilities and predictive performance. Furthermore, the SHAP framework revealed that surgery, N-stage, and T-stage are the primary decision factors influencing the machine learning model’s predictions. Finally, based on the GBM algorithm, we developed an accessible web-based calculator to predict the risk of liver metastasis in PaNETs.

**Conclusion:**

The GBM model excels in predicting the risk of liver metastasis in PaNETs patients, outperforming other machine learning models and providing critical support for developing personalized medical strategies in clinical practice.

## Introduction

Neuroendocrine neoplasms (NENs) are a class of highly heterogeneous tumors, originating from neuroendocrine cells, with the pancreas being a common site of disease (1, 2). Pancreatic neuroendocrine tumors (PaNETs) have a higher incidence and lower diagnostic rate compared to other NENs, and their clinical manifestations are more complex (3). Although PaNETs are rare tumors, accounting for only 1–5% of pancreatic tumors, their incidence rate and clinical detection rate have been on the rise with advances in diagnostic technologies and the widespread popularization of health screenings (4, 5). Although some PaNETs may exhibit a relatively indolent clinical course, it is important to note that these tumors are inherently malignant and also display a wide spectrum of invasiveness (6). A 20-50% of PaNETs patients have distant metastasis at the time of diagnosis, with liver metastasis being the most common, and prognosis significantly worsens once liver metastasis occurs (7, 8). Meanwhile, as PaNETs patients lack typical clinical manifestations and liver metastases are often indistinguishable from other hepatic conditions on radiographic imaging, this further increases the complexity of clinical diagnosis, with most patients already in advanced stages at the time of diagnosis (9, 10). In recent years, increased use of molecular imaging techniques such as PET/CT and SPECT/CT has improved the detection rate of PaNETs and their liver metastases (11). Meanwhile, molecular diagnostic methods, including serum biomarkers and genetic testing, have offered new perspectives for the early detection of liver metastases (12). Moreover, inflammatory biomarkers may emerge as a promising new key tool with potential applications in the diagnosis, treatment response prediction, and prognostic evaluation of neuroendocrine neoplasms (13). Surgical resection remains the treatment option for resectable PaNETs liver metastases, significantly improving survival rates (14). The introduction of targeted therapies and somatostatin analogs (SSA) has markedly enhanced the efficacy of drug treatments (15, 16). In addition, Peptide Receptor Radionuclide Therapy (PRRT), as an emerging treatment for liver metastases, has demonstrated promising prospects (17). Currently, research on predictive models for liver metastases in PaNETs patients remains relatively underexplored. Moreover, most studies have employed only a single type of feature selection method or logistic regression modeling approach (18, 19). Traditional modeling methods (such as logistic regression) impose strict requirements on data distribution and are susceptible to multicollinearity, as well as exhibiting inherent limitations in handling complex, multidimensional data, thereby limiting their broader applicability (20). Therefore, new models for predicting the risk of liver metastasis in PaNETs still need to be developed.

The integration of machine learning with medicine is rapidly transforming healthcare, with advancements in data science driving widespread applications in clinical diagnosis, personalized treatment, and health monitoring (21). Compared to traditional statistical methods, machine learning optimizes algorithms by learning from data, enabling models to make predictions or decisions with a more multidimensional approach to data associations, making it particularly valuable for analyzing complex medical data (22). However, machine learning models are often treated as “black boxes,” making it difficult to comprehend how they predict outcomes or why specific features are crucial to the results (23, 24). Therefore, providing intuitive explanations for machine learning models is essential to facilitate their application in clinical practice. To address this limitation, Lundberg et al. (25) developed the SHapley Additive exPlanations (SHAP) framework in 2017 to assist clinicians in interpreting advanced machine learning models, with the code available as open-source on GitHub.^1^

In this study, we developed 10 machine learning models based on the Surveillance, Epidemiology, and End Results (SEER) database to predict the probability of liver metastasis in PaNETs patients. Subsequently, we used the best-performing machine learning model to develop a web-based calculator to assist clinicians in assessing the risk of liver metastasis in PaNETs patients. This predictive tool provides important references for making personalized clinical decision−making and optimizing healthcare resource allocation.

## Materials and methods

### Patient selection

**FIGURE 1:**
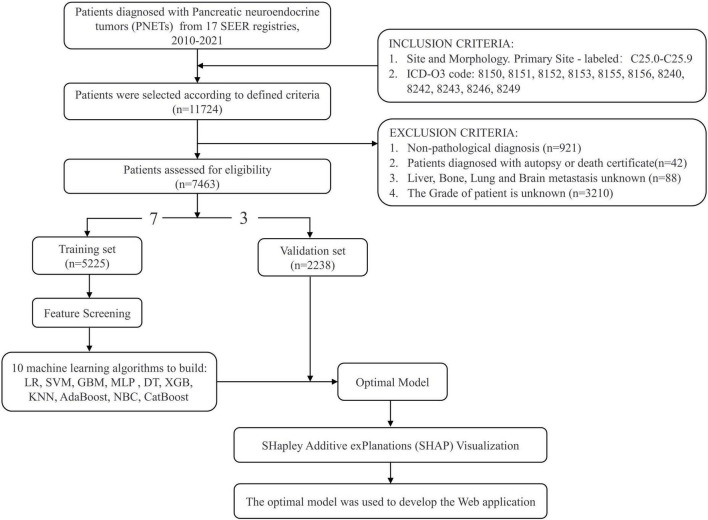
Flowchart of study design and patient screening.

The SEER database is one of the most comprehensive population-based cancer registries in the United States, covering nearly 28% of the U.S. population and providing essential data for investigating complex diseases (26). In this study, we obtained data on patients with PaNETs from the SEER database between 2010 and 2021 (with records sourced from 17 cancer registries), as detailed information on patients’ liver, brain, lung, and bone metastases was not collected in the database until after 2010 (27). Data for this research were acquired by downloading from SEER*Stat software version 8.4.4 in October 2024. The inclusion criteria were as follows: (1) The primary location of pancreatic tumors was classified based on site and morphology codes as C25.0 to C25.9. (2) The following histological/behavioral codes according to the International Classification of Diseases for Oncology, 3rd Edition (ICD-O-3), were used: pancreatic malignant pancreatic endocrine tumor (8,150), insulinoma (8,151), glucagonoma (8,152), gastrinoma (8,153), vipoma (8,155), somatostatinoma (8,156), carcinoid tumor (8,240), malignant enterochromaffin-like cell tumor (8,242), goblet cell carcinoid (8,243), neuroendocrine carcinoma (8,246), and atypical carcinoid tumor (8,249). We excluded the following patients: Patients with non-pathological diagnosis, unknown is the information regarding liver, brain, lung, and bone metastases, unknown grade, and diagnosed with PaNETs only by autopsy or death certificate. A flowchart depicting the study protocol is shown in Figure 1. Since SEER data is publicly available and does not include any identifiable information or personal details, ethical review and informed consent are not necessary.

### Research variables

After filtering the data and excluding missing values, the demographic and clinicopathological variables were obtained, including: year of diagnosis, age at diagnosis (<60, ≥ 60 years old), sex, race (white, black, Asian, other), marital status, annual household income, and location of residence, T-stage, N-stage, tumor size (<2, 2–4, ≥ 4 cm), tumor functional status, primary site (head, body or tails, other), grade (I, II, III), surgery, lymphadenectomy, radiotherapy, chemotherapy, liver metastasis, bone metastasis, lung metastasis and brain metastasis. Marital status was categorized as married, unmarried, separated, divorced, or widowed (SDW). Based on the 2023 Rural-Urban Continuum Codes, the place of residence is classified as either metropolitan county, non-metropolitan, or unknown (28). The tumors were categorized into G1 (≤2%), G2 (2–20%), and G3 (>20%) based on the Ki-67 index (29). In this study, we defined “G1,” “G2,” and “G3” as “I,” “II,” and “III,” respectively, and combined Grades IV and III into a single category (1, 30). The surgeries were categorized into the following four types: None, pancreatectomy and duodenectomy (PD), partial pancreatectomy (PP), and total pancreatectomy (TP).

### Feature selection

The least absolute shrinkage and selection operator (LASSO) is a regularization technique in regression that applies a penalty term to shrink certain regression coefficients, facilitating variable selection and model simplification, while preserving high predictive accuracy (31). The Boruta algorithm is a feature selection method based on Random Forest, which assesses feature importance by creating “shadow variables” for each original variable in the dataset (32). We used the glmnet package in R to perform LASSO regression, setting the key parameter Alpha to 1, and through cross-validation with the cv.glmnet function, we selected lambda.1se to achieve a streamlined model and reduce the risk of overfitting. For the Boruta algorithm, we employed a Random Forest with 500 trees (the default setting in the R “Boruta” package) to obtain robust feature importance measures. We employed the combination of LASSO and Boruta, leveraging Boruta’s global feature assessment capability during feature selection alongside LASSO’s regularization, thereby improving both the accuracy and interpretability of the model.

### Model construction and evaluation

We randomly split the data from the SEER database into training and validation sets in a 7:3 ratio. In this study, we selected 10 well-established supervised machine learning algorithms to build models. These algorithms span linear, tree-based, ensemble, and neural network models, including logistic regression (LR), support vector machine (SVM), gradient boosting machine (GBM), multi-layer perceptron (MLP), random forest (RF), extreme gradient boosting (XGB), k-nearest neighbors (KNN), adaptive boosting (AdaBoost), naive bayes classifiers (NBC), and categorical boosting (CatBoost). Our goal is to address the limitations of model simplicity in current research by comprehensively exploring data features and capturing complex relationships. LR is a linear model widely adopted for binary classification, valued for its interpretability and computational efficiency (33). SVM employs kernel-based boundaries and handles high-dimensional data effectively (34). GBM iteratively trains weak learners to minimize a loss function, thus capturing complex interactions (35). MLP is a feedforward neural network capable of modeling non-linear relationships (36). RF is an ensemble of decision trees that uses bootstrap aggregation to enhance prediction accuracy (37). XGB is a tree-based framework offering efficient and regularized gradient boosting, widely used in medical modeling (38). KNN labels points by their nearest neighbors in feature space, making it widely used in pattern recognition and data mining (39). NBC applies Bayes’ theorem under an independence assumption, which allows it to handle continuous feature values when they occur (40). AdaBoost iteratively reweights training samples to highlight misclassified instances, refining model performance (41). CatBoost is a decision tree gradient boosting algorithm that efficiently handles categorical and ordered features via permutation-driven methods (42). In the training set, given the significant impact of class imbalance on model performance in binary classification, we applied the Synthetic Minority Over-sampling Technique (SMOTE) to resolve the data imbalance issue (43). We optimized hyperparameters by combining grid search with 10-fold CV, partitioning the dataset into 10 subsets so that in each iteration onefold served as the validation set while the remaining nine trained the model, thereby minimizing overfitting risks to the greatest extent possible and enhancing generalizability (44).

We determined the optimal model by evaluating multiple performance metrics, including accuracy, sensitivity, specificity, positive predictive value (PPV), negative predictive value (NPV), F1 score, Kappa score, area under the curve (AUC), and area under the precision-recall (PR) curve (AUPRC). AUC is typically calculated based on the receiver operating characteristic (ROC) curve. However, for imbalanced datasets, AUC may be less effective than the area under the AUPRC in evaluating model performance, so we generated the PR curve and calculated the AUPRC as a complementary metric (45). In addition, we employed decision curve analysis (DCA) to assess the clinical utility of the models. Calibration curves were plotted to compare the predictive performance of the models. Integrated discrimination improvement (IDI) and net reclassification improvement (NRI) to evaluate the improvement in predictive performance of the new model relative to the baseline model (46).

In order to better understand the “black-box” nature of machine learning models, this study employed SHAP to interpret the. The core concept is to calculate each feature’s contribution to the model’s output, providing visual explanations at both global and local levels (47). This approach enhances the transparency of the model’s decision-making process and makes it easier to understand. Furthermore, to promote the clinical adoption and dissemination of the model, we developed an accessible web-based calculator.

### Statistical analysis

In this study, all statistical analyses were performed using R software (version 4.4.1) and Python software (version 3.12). Continuous variables are presented as medians and interquartile ranges (IQR) and compared using the Mann-Whitney U test. Categorical variables are presented as frequencies and percentages (%), and analysis was performed using Fisher’s exact test or Pearson’s chi-square test. The correlation between two variables was analyzed using Spearman’s correlation analysis. The dataset was randomly divided into 70% for training and 30% for testing to develop predictive models. We used the imbalanced-learn library (version 0.12.3) in Python to implement the SMOTE algorithm for oversampling minority class samples. For each minority sample, SMOTE generates synthetic instances by interpolating between its k-nearest neighbors (*k* = 5), thereby effectively addressing the class imbalance problem. Subsequently, 10 machine learning algorithms were used to train the models on the training set. To mitigate overfitting, 10-fold CV was conducted to optimize model parameters during the training process. For interpretability analysis, the SHAP library in Python (version 0.46.0) was applied. *P* < 0.05 (bilateral) was considered statistically significant.

## Results

### Baseline clinical characteristics of patients

**FIGURE 2:**
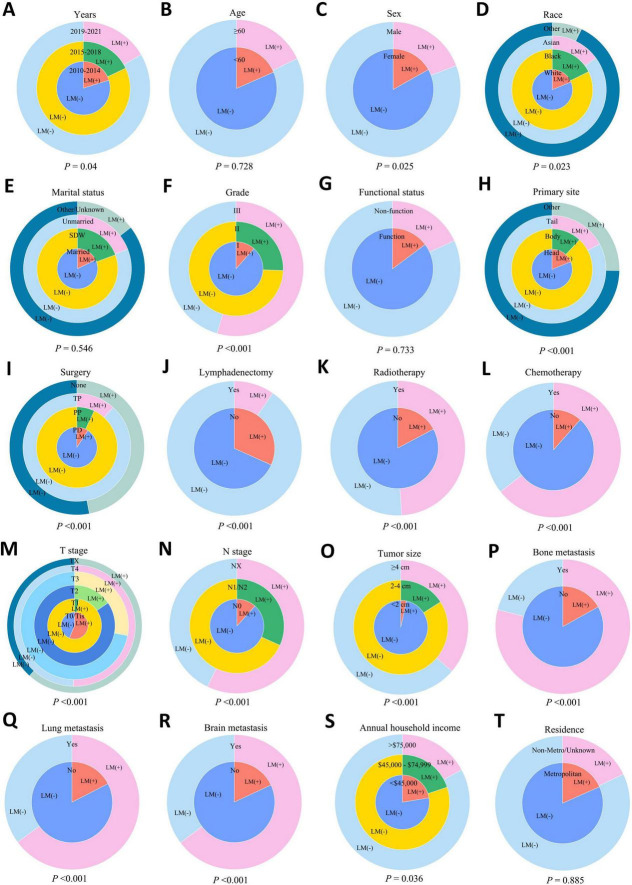
Inset pie charts visualizing the probability of liver metastasis under different clinical and tumor characteristics in PaNETs. **(A)** Years; **(B)** age; **(C)** sex; **(D)** race; **(E)** marital status; **(F)** tumor grade; **(G)** functional status; **(H)** primary site; **(I)** surgery; **(J)** lymphadenectomy; **(K)** radiotherapy; **(L)** chemotherapy; **(M)** T stage; **(N)** N stage; **(O)** tumor size; **(P)** bone metastasis; **(Q)** lung metastasis; **(R)** brain metastasis; **(S)** annual household income; and **(T)** residence. LM (+), with liver metastasis; LM (–), without liver metastasis; SDW, Separated + Divorced + Widowed; PP, Partial pancreatectomy; PD, Pancreatectomy and duodenectomy; TP, Total pancreatectomy. PaNETs, Pancreatic neuroendocrine tumor.

In this study, we include a total of 7,463 patients with PaNETs for detailed retrospective analysis. Of these, 1,356 cases (18.2%) presented with liver metastases, while 6,107 cases (81.8%) did not. Compared to patients without liver metastases, those with liver metastases had a higher proportion of tumors grade (II-III), T-stage (II-IV) and N-stage (N1/2), with 876 cases (64.6%) presenting tumors larger than 4 cm (*P* < 0.001). The incidence of bone, lung, and brain metastases was also significantly higher (*P* < 0.001). In terms of treatment, the liver metastases group had a higher proportion of patients who did not undergo surgery or Lymphadenectomy (*P* < 0.001). The demographic and clinicopathological characteristics of PaNETs patients with and without liver metastases are shown in Table 1 and Figure 2. The subjects were then divided into a training set (*n* = 5,225) and a validation set (*n* = 2,238) at a 7:3 ratio. A total of 4,019 patients (54.3%) were aged 60 years or older, and 4,139 (55.5%) were male. Additionally, most tumors were located in the pancreatic tail (39.2%), with PP as the most common surgical approach (40.4%), while the majority of patients did not receive radiotherapy (97.1%) or chemotherapy (87.4%). No statistically significant differences in demographic or clinicopathological characteristics were observed between the training and validation sets (all *P* > 0.05). Detailed information is provided in Table 2.

**TABLE 1:** Baseline characterization of patients diagnosed as PaNETs patients.

Variables	Without liver metastasis	Without liver metastasis	Total	*P*-value
	(*n* = 6,107), *n* (%)	(*n* = 1,356), *n* (%)	(*n* = 7,463), *n* (%)	
Years				0.04
2010–2014	1,774 (29.0)	448 (33.0)	2,222 (29.8)	
2015–2018	2,270 (37.2)	497 (36.7)	2,767 (37.1)	
2019–2021	2,063 (33.8)	411 (30.3)	2,474 (33.2)	
Age				0.728
< 60	2,805 (45.9)	639 (47.1)	3,444 (46.1)	
≥ 60	3,302 (54.1)	717 (52.9)	4,019 (53.9)	
Sex				0.025
Female	2,765 (45.3)	559 (41.2)	3,324 (44.5)	
Male	3,342 (54.7)	797 (58.8)	4,139 (55.5)	
Race				0.023
White	4,761 (78.0)	1,103 (81.3)	5,864 (78.6)	
Black	658 (10.8)	141 (10.4)	799 (10.7)	
Asian	597 (9.8)	105 (7.7)	702 (9.4)	
Other	91 (1.5)	7 (0.5)	98 (1.3)	
Marital status				0.546
Married	3,891 (63.7)	847 (62.5)	4,738 (63.5)	
SDW	943 (15.4)	227 (16.7)	1,170 (15.7)	
Unmarried	1,002 (16.4)	236 (17.4)	1,238 (16.6)	
Other/Unknown	271 (4.4)	46 (3.4)	317 (4.2)	
Grade				<0.001
I	4,589 (75.1)	602 (44.4)	5,191 (69.6)	
II	1,247 (20.4)	429 (31.6)	1,676 (22.5)	
III	271 (4.4)	325 (24.0)	596 (8.0)	
Functional status				0.733
Function	69 (1.1)	12 (0.9)	81 (1.1)	
Non-function	6,038 (98.9)	1,344 (99.1)	7,382 (98.9)	
Primary site				<0.001
Head	1,063 (27.1)	384 (28.3)	2,037 (27.3)	
Body	1,080 (17.7)	146 (10.8)	1,226 (16.4)	
Tail	2,423 (38.7)	504 (37.2)	2,927 (39.2)	
Other	951 (15.6)	322 (23.7)	1,273 (17.1)	
Surgery				<0.001
PD	1,715 (28.1)	175 (12.9)	1,890 (25.3)	
PP	2,804 (45.9)	214 (15.8)	3,018 (40.4)	
TP	587 (9.6)	78 (5.8)	665 (8.9)	
None	1,001 (16.4)	889 (65.6)	1,890 (25.3)	
Lymphadenectomy				<0.001
No	5,773 (94.5)	753 (55.5)	6,526 (87.4)	
Yes	334 (5.5)	603 (44.5)	937 (12.6)	
Radiotherpy				<0.001
No	5,995 (98.2)	1,248 (92.0)	7,243 (97.1)	
Yes	112 (1.8)	108 (8.0)	220.(2.9)	
Chemotherpy				<0.001
No	5,773 (94.5)	753 (55.5)	6,526 (87.4)	
Yes	334 (5.5)	603 (44.5)	937 (12.6)	
T stage				<0.001
T0/Tis	7 (0.1)	9 (0.7)	16 (0.2)	
T1	2,525 (1.3)	65 (4.8)	2,590 (34.7)	
T2	1,999 (32.7)	368 (27.1)	2,367 (31.7)	
T3	1,221 (20.0)	474 (35.0)	695 (22.7)	
T4	220 (3.6)	223 (16.4)	443 (5.9)	
TX	135 (2.2)	217 (16.0)	352 (4.7)	
N stage				<0.001
N0	4,796 (78.5)	636 (46.9)	5,432 (72.8)	
N1/N2	1,183 (19.4)	549 (40.5)	1,732 (23.2)	
NX	128 (2.1)	171 (12.6)	299 (4.0)	
Tumor size				<0.001
<2 cm	2,495 (40.9)	87 (6.4)	2,582 (34.6)	
2–4 cm	2,073 (33.9)	393 (29.0)	2,466 (33.0)	
≥ 4 cm	1,539 (25.2)	876 (64.6)	2,415 (32.4)	
Bone metastasis				
No	6,075 (99.5)	1,235 (91.1)	7,310 (97.9)	<0.001
Yes	32 (0.5)	121 (8.9)	153 (2.1)	
Lung metastasis				<0.001
No	6,075 (99.5)	1,282 (94.5)	7,349 (98.5)	
Yes	32 (0.5)	74 (5.5)	114 (1.5)	
Brain metastasis				<0.001
No	6,101 (99.9)	1,345 (99.2)	7,446 (99.8)	
Yes	6 (0.1)	11 (0.8)	17 (0.2)	
Annual household income				0.036
< $45,000	83 (1.4)	24 (1.8)	107 (1.4)	
45,000 – $74,999	2,170 (35.5)	538 (39.7)	2,708 (36.3)	
> $75,000	3,854 (63.1)	794 (58.6)	4,648 (62.3)	
Residence				0.885
Metropolitan	3,748 (61.4)	842 (62.1)	4,590 (61.5)	
Non-metro/unknown	2,359 (38.6)	514 (37.9)	2,873 (38.5)	

SDW, Separated + Divorced + Widowed; PP, Partial pancreatectomy; PD, Pancreatectomy and duodenectomy; TP, Total pancreatectomy. PaNETs, Pancreatic neuroendocrine tumor.

**TABLE 2:** Characteristics of PaNETs patients in the training set and the validation set.

Variables	Training set	Validation set	Total	*P*-value
	(*n* = 6,107), *n* (%)	(*n* = 1,356), *n* (%)	(*n* = 7,463), *n* (%)	
Years				0.955
2010–2014	1,557 (29.8)	665 (29.7)	2,222 (29.8)	
2015–2018	1,950 (37.3)	817 (36.5)	2,767 (37.1)	
2019–2021	1,718 (32.9)	756 (33.8)	2,474 (33.2)	
Age				0.561
< 60	2,390 (45.7)	1,054 (47.1)	3,444 (46.1)	
≥ 60	2,825 (54.3)	1,184 (52.9)	4,019 (53.9)	
Sex				0.905
Female	2,336 (44.7)	988 (44.1)	3,324 (44.5)	
Male	2,889 (55.3)	797 (58.8)	4,139 (55.5)	
Race				0.667
White	4,082 (78.1)	1,782 (79.6)	5,864 (78.6)	
Black	581 (11.1)	218 (9.7)	799 (10.7)	
Asian	497 (9.5)	205 (9.2)	702 (9.4)	
Other	65 (1.2)	33 (1.5)	98 (1.3)	
Marital status				0.843
Married	3,302 (63.2)	1,436 (64.5)	4,738 (63.5)	
SDW	816 (15.6)	354 (15.8)	1,170 (15.7)	
Unmarried	890 (17.0)	348 (15.5)	1,238 (16.6)	
Other/unknown	271 (4.2)	100 (4.5)	317 (4.2)	
Grade				0.138
I	3,602 (68.9)	1,589 (71.0)	5,191 (69.6)	
II	1,216 (23.3)	460 (20.6)	1,676 (22.5)	
III	407 (7.8)	189 (8.4)	596 (8.0)	
Functional status				0.171
Function	49 (0.9)	32 (1.4)	81 (1.1)	
Non-function	5,176 (99.1)	2,206 (98.6)	7,382 (98.9)	
Primary site				0.966
Head	1,424 (27.3)	613 (27.4)	2,037 (27.3)	
Body	869 (16.6)	357 (16.0)	1,226 (16.4)	
Tail	2,056 (39.3)	871 (38.9)	2,927 (39.2)	
Other	876 (16.68)	397 (17.7)	1,273 (17.1)	
Surgery				0.975
PD	1,339 (25.6)	551 (24.6)	1,890 (25.3)	
PP	2,105 (40.3)	913 (40.8)	3,018 (40.4)	
TP	470 (9.6)	195 (8.7)	665 (8.9)	
None	1,311 (25.1)	579 (25.9)	1,890 (25.3)	
Lymphadenectomy				0.153
No	1,972 (37.7)	898 (40.1)	2,870 (38.5)	
Yes	3,253 (62.3)	1,340 (59.9)	4,593 (61.5)	
Radiotherpy				0.326
No	5,081 (97.2)	2,162 (96.6)	7,243 (97.1)	
Yes	144 (2.8)	76 (3.4)	220.(2.9)	
Chemotherpy				0.9
No	4,575 (87.6)	1,951 (87.2)	6,526 (87.4)	
Yes	650 (12.4)	287 (12.8)	937 (12.6)	
T stage				0.786
T0/Tis	10 (0.2)	6 (0.3)	16 (0.2)	
T1	1,777 (34.0)	813 (36.3)	2,590 (34.7)	
T2	1,693 (32.4)	674 (30.1)	2,367 (31.7)	
T3	1,187 (22.7)	508 (22.7)	695 (22.7)	
T4	317 (6.1)	126 (5.6)	443 (5.9)	
TX	241 (4.6)	111 (5.0)	352 (4.7)	
N stage				0.933
N0	3,787 (72.5)	1,645 (73.5)	5,432 (72.8)	
N1/N2	1,227 (23.5)	505 (22.6)	1,732 (23.2)	
NX	211 (4.0)	88 (3.9)	299 (4.0)	
Tumor size				0.289
<2 cm	1,769 (33.9)	813 (36.3)	2,582 (34.6)	
2–4 cm	1,760 (33.7)	706 (31.5)	2,466 (33.0)	
≥ 4 cm	1,696 (32.5)	719 (32.1)	2,415 (32.4)	
Liver metastasis				0.999
No	4,275 (81.8)	1,832 (81.9)	6,107 (81.8)	
Yes	950 (18.2)	406 (18.1)	1,356 (18.2)	
Bone metastasis				0.042
No	5,132 (98.2)	2,178 (97.3)	7,310 (97.9)	
Yes	93 (1.8)	60 (2.7)	153 (2.1)	
Lung metastasis				0.971
No	5,144 (98.4)	2,205 (98.5)	7,349 (98.5)	
Yes	81 (1.6)	33 (1.5)	114 (1.5)	
Brain metastasis				0.539
No	5,211 (99.7)	2,235 (99.9)	7,446 (99.8)	
Yes	14 (0.3)	3 (0.1)	17 (0.2)	
Annual household income				0.993
< $45,000	73 (1.4)	34 (1.5)	107 (1.4)	
45,000 – $74,999	1,902 (36.4)	806 (36.0)	2,708 (36.3)	
> $75,000	3,250 (62.2)	1,398 (62.5)	4,648 (62.3)	
Residence				0.477
Metropolitan	3,237 (62.0)	1,353 (60.5)	4,590 (61.5)	
Non-metro/unknown	1,988 (38.0)	885 (39.5)	2,873 (38.5)	

SDW, Separated + Divorced + Widowed; PP, Partial pancreatectomy; PD, Pancreatectomy and duodenectomy; TP, Total pancreatectomy. PaNETs, Pancreatic neuroendocrine tumor.

### Correlation analysis and predictor screening

**FIGURE 3:**
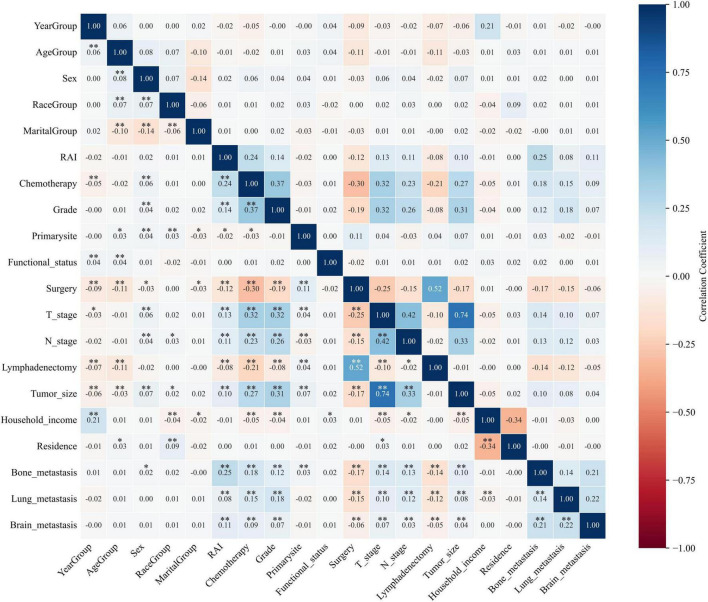
Results of Spearman’s correlation analysis for each variable. *P < 0.05; **P < 0.01.

**FIGURE 4:**
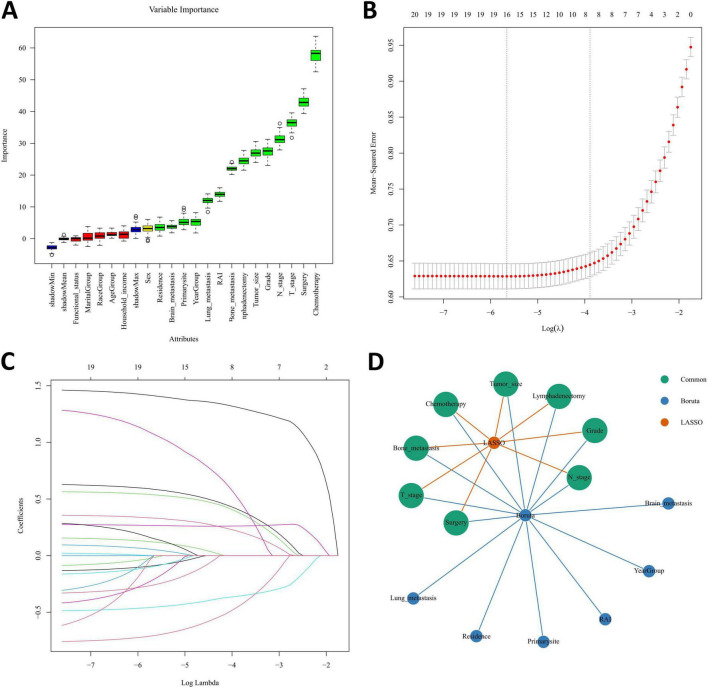
Predictor screening results. **(A)** Boruta; **(B)** LASSO cross-validation curve; **(C)** variable coefficient diagram of LASSO regression model; **(D)** common predictors between Boruta and LASSO.

In order to assess the strength and direction of relationships between variables, correlation analysis is commonly used. In the present research, Spearman’s correlation analysis was performed to assess the independence between data features, and the results were visualized in a correlation heatmap (Figure 3). The results indicate that no severe collinearity was present, as all correlation coefficients were below 0.80, thereby ensuring the reliability of the predictor screening process. Boruta, an extension of the RF algorithm, identifies the most relevant features by iteratively comparing the importance of real features with that of randomized shadow feature. We employed the Boruta algorithm to identify 14 key factors, including year of diagnosis, residence, T-stage, N-stage, tumor size, primary site, grade, surgery, lymphadenectomy, radiotherapy, chemotherapy, bone metastasis, lung metastasis, and brain metastasis (Figure 4A). In comparison, LASSO regression, a shrinkage method, selects variables and adjusts complexity through an optimization function with a penalty term. In this study, lambda.1se was identified as the optimal value, and the eight key variables selected through LASSO regression included T-stage, N-stage, tumor size, grade, surgery, lymphadenectomy, chemotherapy, and bone metastasis (Figures 4B,C). Subsequently, a common subset of variables was identified through a comparative analysis of the feature selection results from the Boruta algorithm and LASSO regression. The selected features were ultimately used for model construction, including T-stage, N-stage, tumor size, grade, surgery, lymphadenectomy, chemotherapy, and bone metastasis (Figure 4D).

### Model performance

**FIGURE 5:**
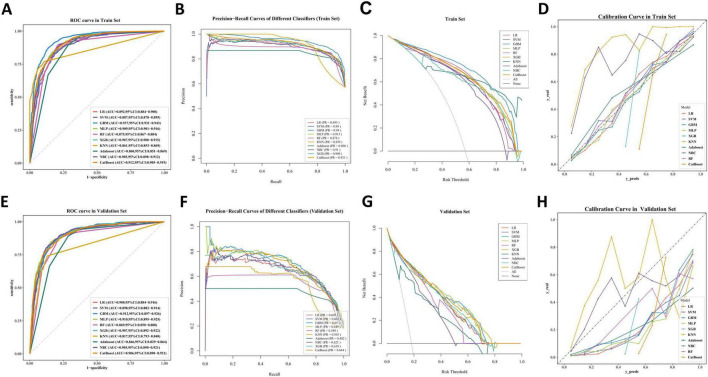
The performance and comparison of 10 different predictive models. **(A)** The training set ROC curve; **(B)** the training set PR curves; **(C)** the training set DCA curves; **(D)** the training set calibration curves. **(E)** The validation set ROC curve; **(F)** the validation set PR curves; **(G)** the validation set DCA curves; **(H)** the validation set calibration curves.

**FIGURE 6:**
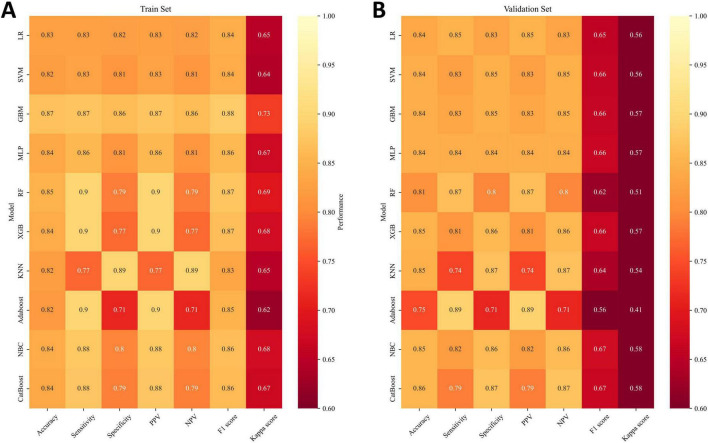
Prediction performance of different models. **(A)** training set; **(B)** validation set. PPV, Positive predictive value; NPV, Negative predictive value.

To obtain the optimal predictive model, we compared the performance of 10 machine learning algorithms and validated them on the validation set. As shown in Figures 5A,E), the GBM algorithm achieved higher AUC values compared to the other nine models, with training set (AUC = 0.937, 95% CI: 0.931–0.943) and validation set (AUC = 0.912, 95% CI: 0.897–0.926). The PR curve indicates that the GBM model achieves a higher AUPRC compared to the other 9 models, with training set (AUPRC = 0.94) and validation set (AUPRC = 0.65) (Figures 5B,F). The DCA curves demonstrate that the GBM model exhibits superior clinical decision-making ability and practical predictive performance in both the training and test sets compared to other models (Figures 5C,G). The calibration curves of different machine learning algorithms indicate that the GBM algorithm shows the highest consistency with the ideal prediction curve in both the training and test sets (Figures 5D,H). Heatmap analysis offers a comprehensive, clear, intuitive, and easily interpretable approach, making it ideal for multidimensional evaluations of model performance. In this study, we employed a heatmap to compare each model’s accuracy, sensitivity, specificity, positive predictive value, negative predictive value, F1 score, and Kappa value across the training and validation sets (Figures 6A,B). In the validation set, NRI and IDI analyses showed that the GBM model outperformed most models in terms of reclassification and overall discrimination capabilities (Supplementary Table 1). After a comprehensive evaluation of the performance of 10 models in the training and validation sets, we identified the GBM model as the best performer in predicting liver metastases in PaNETs patients, designating GBM as the optimal model.

### Interpretability analysis

**FIGURE 7:**
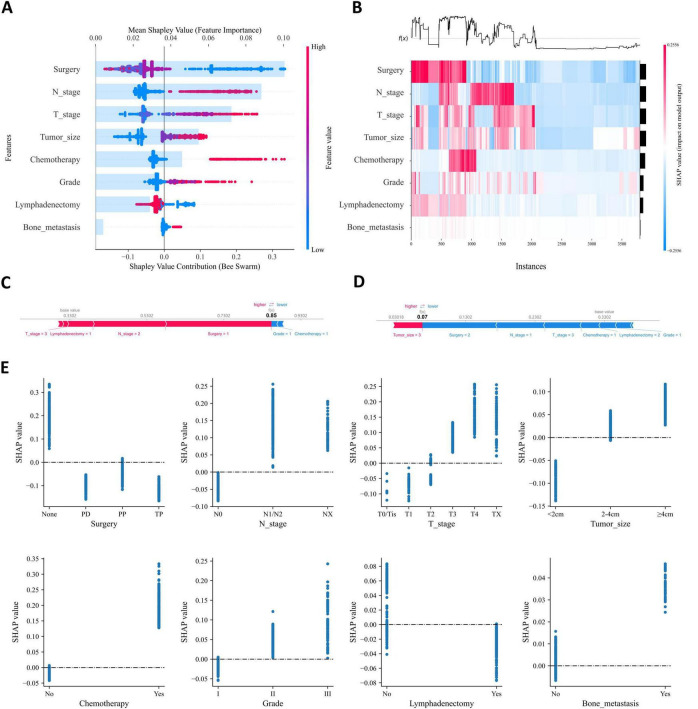
Interpretability analysis of GBM models. **(A)** The SHAP summary plots displaying the importance ranking of features; **(B)** the SHAP heatmap clusters hierarchically based on SHAP values; **(C)** the SHAP force plot for PaNETs patients with liver metastasis; **(D)** the SHAP force plot for PaNETs patients without liver metastasis; **(E)** SHAP dependence plot. Each dependence plot illustrates how a single feature affects the model’s output, with each point representing a patient. Features with SHAP values greater than zero push the decision towards the liver metastasis category.

We applied SHAP framework to interpretation of the GBM model. In SHAP analysis, higher feature SHAP values generally indicate an increased likelihood of the target event. Figure 7A shows all risk factors evaluated using mean absolute SHAP values, with surgery ranked as the most important variable, followed by N-stage, T-stage, tumor size, chemotherapy, grade, lymphadenectomy, and bone metastasis, and illustrating how these factors influence liver metastasis. The SHAP heatmap performs hierarchical clustering of patients based on SHAP values, visually highlighting the distribution of PaNETs patients with and without liver metastasis, where red represents high-probability cases of liver metastasis and colorless or blue indicates cases with no liver metastasis or low probability (Figure 7B). The combination of different variables influences patient prognosis. To improve the understanding of the model’s decision-making on an individual level, we provide two representative samples: one from a PaNETs patient with liver metastasis and another from a patient without liver metastasis (Figures 7C,D). Additionally, the SHAP dependence plot (Figure 7E) illustrates how individual features affect the model’s predicted output and visualizes the changes in their attribution importance as the feature values vary. For example, in PaNETs patients who did not undergo surgical treatment, have higher tumor stages, and larger tumor diameters, the SHAP values are greater than zero, driving the model’s predictions toward the liver metastasis category. Through the visualization of the SHAP values for these samples, we can assess how each feature influences the model’s predictions for these specific cases.

### Web calculator

**FIGURE 8:**
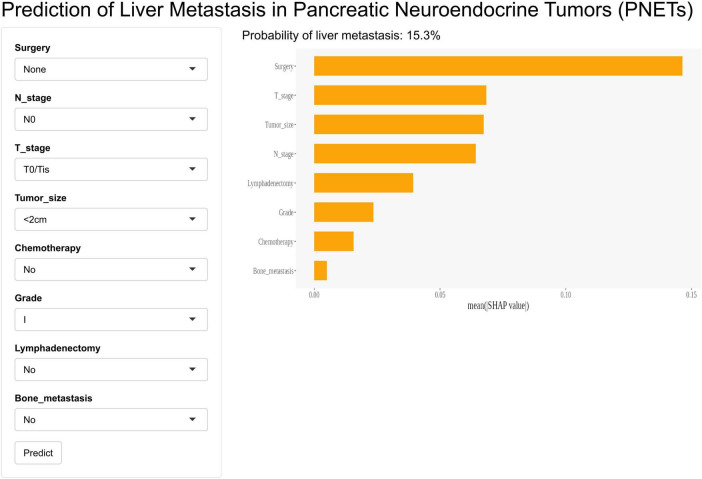
Web calculator for predicting liver metastasis in PaNETs patients (accessible at: https://bijinzhe.shinyapps.io/pnet_lm_shiny/).

In this study, we developed a web-based calculator based on the GBM model to predict liver metastasis in PaNETs patients, aiming to facilitate clinical adoption and dissemination. The image of the web calculator is presented in Figure 8. Clinicians can calculate the probability of liver metastasis in PaNETs patients by entering relevant clinical and pathological information into the web calculator. The web calculator can be conveniently accessed online through the following link^2^.

## Discussion

Distant metastasis is a critical factor affecting the prognosis of PaNETs patients, with previous studies showing a median survival time of 24 months for those with distant metastases (1, 48). When PaNETs patients experience distant metastases, research has confirmed that the liver is the most common target organ (7). Therefore, it is crucial to promptly identify and predict the risk of liver metastases in PaNETs patients. However, no studies have applied interpretable machine learning to predict liver metastasis in PaNETs patients to date. To fill this gap, this study leveraged the SEER database to construct a personalized, accurate, and reliable predictive model for PaNETs liver metastases using multiple machine learning algorithms. In addition, the SHAP framework was utilized to thoroughly investigate variable importance and underlying impact mechanisms, and a web-based online calculator was created to facilitate the clinical adoption and dissemination of the model.

In this study, we applied a combined approach of the Boruta algorithm and LASSO regression to identify key predictive factors, ensuring accurate feature selection and model stability. As a result, the identified features included T-stage, N-stage, tumor size, grade, surgery, lymphadenectomy, chemotherapy, and bone metastasis. We then constructed and comprehensively evaluated the predictive performance of 10 robust machine learning algorithms based on the selected features, identifying GBM as the optimum model for predicting liver metastases in PaNETs patients. The GBM model demonstrated the highest AUC values, achieving 0.937 on the training set and 0.912 on the validation set, as well as the highest AUPRC, with 0.94 on the training set and 0.65 on the validation set. We employed SMOTE to address the issue of data imbalance, as only 18.2% of patients experienced liver metastases. Despite this, the calibration curve still showed slight deviations. However, the GBM model demonstrated a more precise calibration curve and delivered better net benefits compared to the other nine machine learning models.

This research employed the SHAP framework to generate global and local explanations for the machine learning models, enhancing its interpretability and visual transparency. By leveraging SHAP values, we assessed the impact of each factor and observed, through variable importance visualizations, that all factors contributed to the model’s performance (Figure 7A). In this study, surgery was identified as the most critical variable for predicting liver metastases in PaNETs patients. Surgical resection is the only curative treatment for PaNETs patients and is therefore the preferred option for most patients with localized PaNETs (49). Studies have shown that patients undergoing surgical resection of primary tumors and liver metastases have significantly higher survival rates compared to those who do not undergo surgery (50, 51). This may be attributed to surgical resection reducing circulating tumor cells and micrometastatic burden (52). Therefore, surgery plays a vital role in the treatment of PaNETs patients, effectively reducing the risk of liver metastases and improving patient prognosis. The N stage is the second most important variable after surgery. Lymph node metastasis is not only an indicator of local dissemination but is also commonly associated with an increased risk of cancer spreading to distant organs, thereby profoundly influencing treatment strategies and patient prognosis, and this correlation has been well demonstrated in studies on other tumor types (53, 54). Therefore, greater attention should be paid to metastases in the liver and other regions in patients with positive lymph nodes. This study identified T stage and tumor size as the third and fourth most important variables for liver metastasis in PaNETs, and revealed a close relationship between larger tumor size and higher T stage. The larger the tumor and the higher the T-stage, indicating greater invasiveness into surrounding organs and blood vessels, which significantly increases the likelihood of liver metastases (55). Previous research has demonstrated that chemotherapy may enhance the metastasis of malignant tumors by promoting the expression of metastasis-associated genes, inducing the formation of a pro-metastatic tumor microenvironment, and increasing the secretion of exosomes that drive metastasis (56–58). This indicates that while chemotherapy may lead to tumor shrinkage, it could also increase the risk of metastasis. In PaNETs, bone metastases are uncommon compared to liver metastases and lung metastases. Research indicates that the presence of bone metastases is associated with the progression of liver metastases, impacting overall survival and treatment outcomes (59). The precise mechanisms underlying this relationship in PaNETs require further investigation. Although our study did not identify gender as a significant predictor of liver metastasis in PaNETs, a recent systematic review indicates that gender disparities are gaining increasing attention in the clinical management and prognostic evaluation of PaNETs (60). This may be attributed to the specific focus on liver metastasis or population heterogeneity in our study, underscoring the need for future large-scale research to further explore gender-related biological or clinical disparities in PaNETs. In addition, SHAP demonstrated superior performance compared to the Local Interpretable Model-Agnostic Explanations (LIME) method in both global and individual explanation tasks, with LIME exhibiting lower consistency in individual analyses (61). Accordingly, we utilized SHAP force plots to present two representative personalized samples (Figures 7C,D), further enhance the interpretability of the machine learning model.

From a clinical perspective, this study is of great significance for improving early detection and intervention strategies for liver metastases in patients with PaNETs. We recommend that this tool be integrated into hospital electronic health record systems and routine clinical workflows in the future so that clinicians can utilize it in real time during initial consultation, throughout treatment, and during follow-up. By inputting patient-related variables into a web-based calculator, personalized probabilities of liver metastasis can be generated, thus enabling early identification and targeted management of high-risk patients. It is noteworthy that although SHAP values provide a high degree of interpretability for the model, clinicians must be cautious of over-relying on its outputs. Therefore, we recommend combining model predictions with clinical judgment and patients’ longitudinal follow-up data to ensure the accuracy and clinical applicability of risk assessments.

Nevertheless, this study has some limitations. Firstly, the data for this study were derived from a retrospective analysis of the SEER database, which may introduce concerns such as data quality issues, information bias, and selection bias (62). Although recent geopolitical developments have changed the access conditions for the SEER database, it must be emphasized that our study utilized a complete dataset obtained prior to April 2025, thereby ensuring its integrity and validity. Secondly, the limitations of the SEER database pose challenges in obtaining additional relevant information, such as detailed data on Ki-67 index, SSA, targeted therapy, radiotherapy and chemotherapy. Notably, Ki-67 index and SSA are critical factors in the management and prognosis of PaNETs (63, 64). Thirdly, our data were sourced from the SEER database without external validation using data from other hospitals. The performance of machine learning models may vary among patients from different regions and hospitals. In future studies, we plan to incorporate prospective designs and integrate multi-center data, including but not limited to imaging data, genomic information, and other detailed clinical data, for model validation and testing. The aim of these improvements is to enhance the model’s robustness and generalizability, thereby offering more personalized and precise treatment strategies for PaNETs patients.

## Conclusion

In summary, we have successfully developed an interpretable machine learning model to predict the risk of liver metastasis in PaNETs patients based on clinical data. The final GBM model demonstrated superior and reliable predictive performance. By utilizing our web-based calculator, clinicians can formulate and dynamically adjust personalized clinical decision-making strategies, thereby improving patient prognosis.

## Data Availability

The raw data supporting the conclusions of this article will be made available by the authors, without undue reservation.

## Footnotes

1
https://github.com/slundberg/shap

2
https://bijinzhe.shinyapps.io/pnet_lm_shiny/
