# Supplementary material for: Predicting liver metastasis in pancreatic neuroendocrine tumors with an interpretable machine learning algorithm: a SEER-based study
Source: Front Med (Lausanne). 2025 May 1;12:1533132. doi: 10.3389/fmed.2025.1533132 (PMC12078274; doi:10.3389/fmed.2025.1533132)
Supplement: Supplementary file 1 [file Table_1.docx]

Supplementary TABLE 1 NRI and IDI comparison of various machine learning models in validation set.

| **New model-Baseline model** | **NRI** | **P value** | **IDI** | **P value** |
| --- | --- | --- | --- | --- |
| SVM-LR | 0.0115(-0.0147~0.0377) | 0.38993 | 0.0257(0.0204~0.031) | <0.0001 |
| GBM-LR | 0.0553(0.0153~0.0953) | 0.00668 | 0.048(0.0365~0.0595) | <0.0001 |
| MLP-LR | 0.0694(0.0309~0.108) | 0.00041 | 0.0405(0.0307~0.0504) | <0.0001 |
| RF-LR | 0.1279(0.0738~0.1819) | <0.0001 | -0.0044(-0.0225~0.0137) | 0.63638 |
| XGB-LR | -1.2016(-1.2646~-1.1387) | <0.0001 | -0.4831(-0.5079~-0.4584) | <0.0001 |
| KNN-LR | -0.2159(-0.2745~-0.1574) | <0.0001 | -0.0723(-0.1105~-0.0342) | 0.0002 |
| Adaboost-LR | -0.1064(-0.1601~-0.0526) | 0.0001 | 0.0776(0.0551~0.1001) | <0.0001 |
| NBC-LR | -0.0561(-0.1077~-0.0046) | 0.03274 | 0.0572(0.0364~0.078) | <0.0001 |
| CatBoost-LR | -0.6689(-0.7224~-0.6154) | <0.0001 | -0.3758(-0.3959~-0.3558) | <0.0001 |
| GBM-SVM | 0.0422(0.0014~0.083) | 0.0428 | 0.0223(0.0101~0.0346) | 0.00036 |
| MLP-SVM | 0.0579(0.0204~0.0955) | 0.0025 | 0.0148(0.0034~0.0262) | 0.01096 |
| RF-SVM | 0.109(0.0589-0.1591) | 0.00002 | -0.0301(-0.0479~-0.0123) | 0.00092 |
| XGB-SVM | -1.2131(-1.278~-1.1483) | <0.0001 | -0.5088(-0.5347~-0.4829) | <0.0001 |
| KNN-SVM | -0.2247(-0.2852~-0.1642) | <0.0001 | -0.098(-0.1379~-0.0582) | <0.0001 |
| Adaboost-SVM | -0.1179(-0.1755~-0.0603) | 0.00006 | 0.0519(0.029~0.0748) | 0.00001 |
| NBC-SVM | -0.0712(-0.1233~-0.0191) | 0.00741 | 0.0315(0.0099~0.0531) | 0.00431 |
| CatBoost-SVM | -0.6804(-0.735~-0.6258) | <0.0001 | -0.4015(-0.4228~-0.3802) | <0.0001 |
| MLP-GBM | 0.013(-0.0177~0.0437) | 0.40627 | -0.0075(-0.0148~-2e-04) | 0.0436 |
| RF-GBM | 0.0608(0.0137~0.1078) | 0.01134 | -0.0524(-0.0658~-0.039) | <0.0001 |
| XGB-GBM | -1.2581(-1.3254~-1.1908) | <0.0001 | -0.5312(-0.5572~-0.5051) | <0.0001 |
| KNN-GBM | -0.2702(-0.3329~-0.2074) | <0.0001 | -0.1204(-0.1602~-0.0806) | <0.0001 |
| Adaboost-GBM | -0.1655(-0.2203~-0.1108) | <0.0001 | 0.0296(0.0083~0.0508) | 0.00634 |
| NBC-GBM | -0.109(-0.1631~-0.0549) | 0.00008 | 0.0092(-0.015~0.0333) | 0.45682 |
| CatBoost-GBM | -0.7254(-0.7795~-0.6712) | <0.0001 | -0.4239(-0.4451~-0.4027) | <0.0001 |
| RF-MLP | 0.0519(0.0079~0.0959) | 0.02087 | -0.0449(-0.0604~-0.0293) | <0.0001 |
| XGB-MLP | -1.2711(-1.3368~-1.2053) | <0.0001 | -0.5236(-0.5489~-0.4984) | <0.0001 |
| KNN-MLP | -0.2618(-0.3212~-0.2024) | <0.0001 | -0.1129(-0.1519~-0.0739) | <0.0001 |
| Adaboost-MLP | -0.1813(-0.2357~-0.1269) | <0.0001 | 0.0371(0.0151~0.059) | 0.00093 |
| NBC-MLP | -0.1022(-0.1545~-0.0499) | 0.00013 | 0.0167(-0.0082~0.0415) | 0.18904 |
| CatBoost-MLP | -0.7384(-0.7965~-0.6803) | <0.0001 | -0.4163(-0.4367~-0.396) | <0.0001 |
| XGB-RF | -1.3123(-1.3883~-1.2362) | <0.0001 | -0.4788(-0.5052~-0.4523) | <0.0001 |
| KNN-RF | -0.3209(-0.3807~-0.261) | <0.0001 | -0.068(-0.1118~-0.0241) | 0.00238 |
| Adaboost-RF | -0.2227(-0.2789~-0.1665) | <0.0001 | 0.082(0.0615~0.1024) | <0.0001 |
| NBC-RF | -0.1517(-0.2031~-0.1004) | <0.0001 | 0.0616(0.0321~0.091) | 0.00004 |
| CatBoost-RF | -0.784(-0.8531~-0.7148) | <0.0001 | -0.3714(-0.3941~-0.3488) | <0.0001 |
| KNN-XGB | 0.7786(0.6868~0.8705) | <0.0001 | 0.4108(0.3686~0.453) | <0.0001 |
| Adaboost-XGB | 1.0895(1.0227~1.1564) | <0.0001 | 0.5607(0.5287~0.5928) | <0.0001 |
| NBC-XGB | 1.1271(1.04~1.2142) | <0.0001 | 0.5403(0.5023~0.5783) | <0.0001 |
| CatBoost-XGB | 0.5327(0.4837~0.5817) | <0.0001 | 0.1073(0.102~0.1126) | <0.0001 |
| Adaboost-KNN | 0.133(0.0637~0.2023) | 0.00017 | 0.1499(0.1024~0.1974) | <0.0001 |
| NBC-KNN | 0.205(0.1426~0.2675) | <0.0001 | 0.1295(0.0885~0.1706) | <0.0001 |
| CatBoost-KNN | -0.4083(-0.4742~-0.3424) | <0.0001 | -0.3035(-0.3438~-0.2632) | <0.0001 |
| NBC-Adaboost | 0.0663(0.0063~0.1262) | 0.03036 | -0.0204(-0.0555~0.0147) | 0.25494 |
| CatBoost-Adaboost | -0.5588(-0.6227~-0.4948) | <0.0001 | -0.4534(-0.4823~-0.4245) | <0.0001 |
| CatBoost-NBC | -0.5963(-0.6527~-0.54) | <0.0001 | -0.433(-0.4671~-0.3989) | <0.0001 |

Abbreviations: NRI: Net Reclassification Improvement; IDI: Integrated Discrimination Improvement.
